# The clinicopathological characteristics and prognosis of young patients with chondrosarcoma of bone

**DOI:** 10.3389/fsurg.2022.926008

**Published:** 2022-09-05

**Authors:** Tao Xie, Yuanyuan Sun, Xiao Han, Jian Zhang

**Affiliations:** ^1^ Department of Orthopedic Surgery, Affiliated Hangzhou First People’s Hospital, Zhejiang University School of Medicine, Hangzhou, China; ^2^ Department of Neurology, The Second Affiliated Hospital, Zhejiang University School of Medicine, Hangzhou, China

**Keywords:** clinicopathological characteristics, chondrosarcoma of bone, young patients, prognosis, survival predictor

## Abstract

**Purpose:**

Clinicopathologic characteristics and treatment outcomes for young patients (less than 40 years) with chondrosarcoma of bone are rarely documented. The purpose of this study is to determine the clinicopathological characteristics and identify the survival predictors for this rare population.

**Patients and Methods:**

We used the Surveillance, Epidemiology, and End Results (SEER) database to identify young patients with chondrosarcoma of bone between 1973 and 2016. Univariate and multivariate Cox regression analyses were conducted to determine the independent risk factors. Kaplan-Meier method was used to intuitively show the survival difference stratified by different treatments.

**Results:**

A total of 1312 eligible young patients with chondrosarcoma of bone were analyzed this study. The mean age at diagnosis was 28.5 ± 0.2 years old (ranging from 1 to 40 years). 51.1% of cases were located in the extremity. More than two-thirds of patients (71.4%) were high grade. The majority of the patients (92.0%) received surgery, only 11.8% of patients received radiotherapy, and only 10.4% of patients received chemotherapy. The 5-year overall survival (OS) and cancer-specific survival (CSS) rates of this cohort were 88.5% and 89.1%, respectively. According to the results of multivariate analysis, nine variables were significantly correlated with OS and CSS, including gender, year of diagnosis, tumor site, tumor grade, tumor subtype, distant metastasis, tumor size, surgery, and chemotherapy.

**Conclusion:**

Young patients with chondrosarcoma of bone experienced better prognosis. Surgery was significantly correlated with increased survival, while chemotherapy was significantly correlated with decreased survival. Radiotherapy was not a meaningful survival predictor of young patients with chondrosarcoma of bone. Prospective clinical trials are needed in the future to determine the effect of radiotherapy and chemotherapy on prognosis of those patients.

## Introduction

Chondrosarcoma is the second most frequent primary malignant bone tumor characterized by the formation of cartilage (1). Chondrosarcoma occurs predominantly in patients aged 50–70 years (2, 3). Previous studies reported that age was an important survival predictor of chondrosarcoma and young patients may have better prognosis (4). The clinicopathologic characteristics and treatment outcomes may be different in young patients with chondrosarcoma. At present, treatment strategies of chondrosarcoma contain surgical resection, chemotherapy and radiotherapy. Therapeutic surgical approach is still the dominant treatment. Although chondrosarcoma respond poorly to chemotherapy and radiotherapy (5), both treatments are still used in this tumor. With the development of chemoradiotherapy, some scholars have reconsidered the clinical significance of chemotherapy and radiotherapy in the treatment of chondrosarcoma (6, 7).

The clinical features, survival time, and risk factors of survival among all chondrosarcomas are widely reported (8, 9). However, as far as we know, there were limited studies that evaluated clinicopathologic characteristics and treatment outcomes for young patients with chondrosarcoma of bone. Moreover, optimal treatments for young patients with chondrosarcoma of bone remain unknown. Therefore, our study aims to investigate associations between clinical characteristics, treatments and survival among young patients with chondrosarcoma of bone, based on the Surveillance, Epidemiology, and End Results (SEER) database.

## Materials and methods

### Patient population

We collected information on young patients with chondrosarcoma of bone from the research plus database of SEER*Stat 8.3.9 from 1973 to 2016. The SEER database (November 2021 submission) collects cancer information from 18 locations, covering 30 percent of the U.S. population. The SEER database does not contain patient' identification information. Thus, this study is exempt from clinical ethical review.

**Figure 1:**
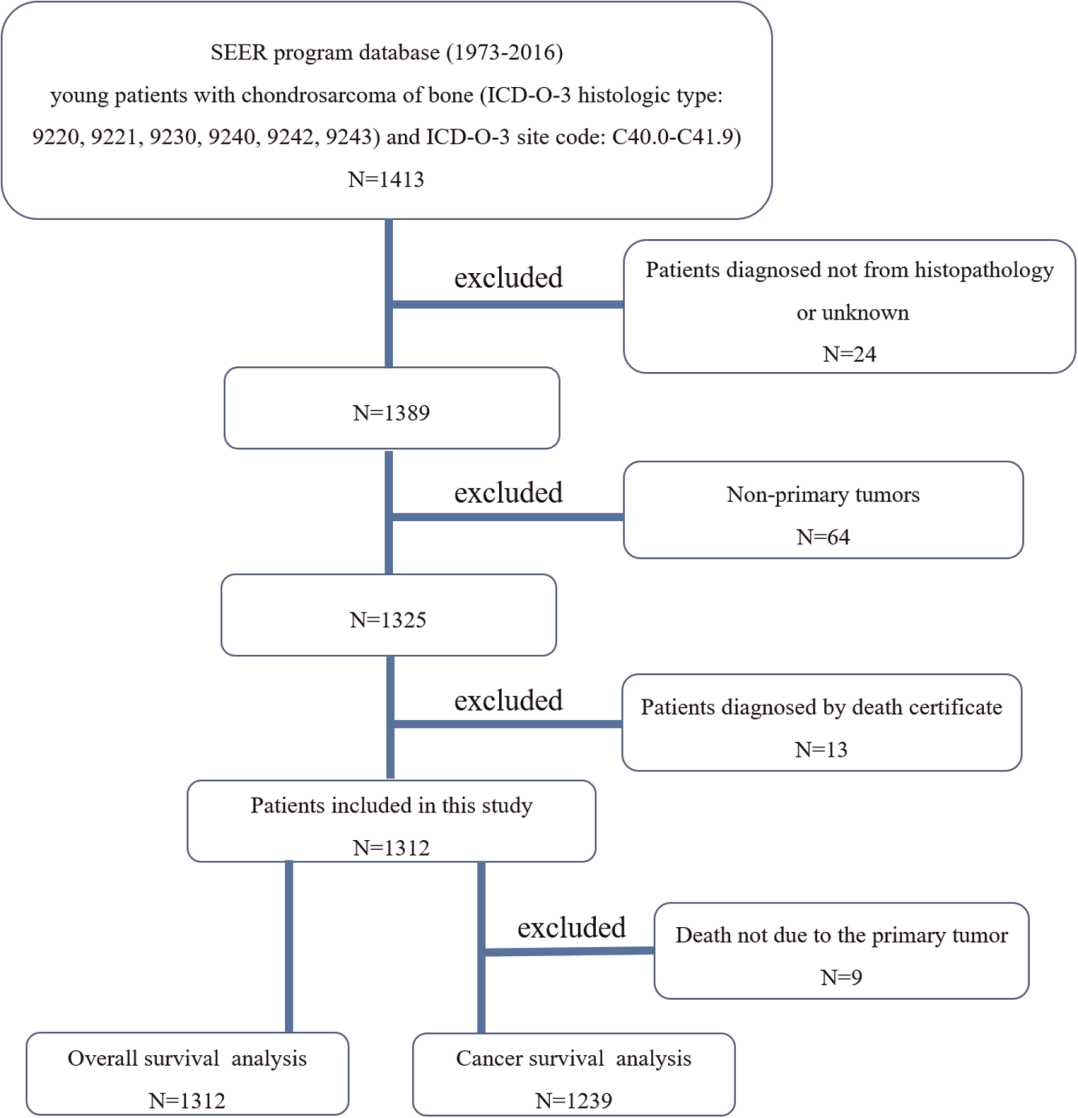
The flow chart for nselection of study population. SEER, Surveillance, Epidemiology, and End Results; ICD-O-3, international classification of diseases for oncology, 3rd edition.).

According to the International Classification of Diseases for Oncology, 3rd edition (ICD-O-3), we conducted the case-listing procedure to include patients with chondrosarcoma (ICD-O-3 histologic type: 9220, 9221, 9230, 9240, 9242, 9243) of bone (ICD-O-3 site code: C40.0-C41.9). Only patients aged less than 40 years old were included for analysis. Twenty-four patients were excluded due to the diagnosis only based on the clinical presentation or the imaging. Sixty-four patients were excluded because of non-primary tumors. Thirteen patients diagnosed by death certificate were excluded. The patient selection flowchart was shown in Figure 1. The variables including race, gender, age, year of diagnosis, tumor site, tumor grade, tumor subtype, distant status, tumor size, surgery of primary site, radiotherapy of primary site, systemic chemotherapy, marital status, death reasons, survival months, and vital status were extracted from the SEER database. Other tumor subtypes include juxtacortical chondrosarcoma, chondroblastoma, malignant, mesenchymal chondrosarcoma, clear cell chondrosarcoma, and dedifferentiated chondrosarcoma. We respectively defined overall survival (OS) and cancer-specific survival (CSS) as the time from diagnosis till death due to any reason and due to chondrosarcoma, which were the main end points of the study.

### Statistical analyses

We applied the SPSS 22.0 software to perform all statistical analyses and generate graphics. We first performed univariate Cox regression analysis to screen risk factors with *p* < 0.1 for multivariate Cox regression analysis. In this way, some variables that may be meaningless can be filtered out and the results are more reliable. Then multivariate Cox regression analyses were conducted to determine the independent risk factors. We also calculated the hazard ratios (HRs) and their 95% confidence intervals (95% CIs) to indicate the impact of each risk factor on survival. Kaplan-Meier method was used to compare survival across strata accompanied by Log-rank test procedure. Statistical results were considered of significance if two-sided *p* value was <0.05.

## Results

### Clinicopathological characteristics

Our study cohort included a total of 1,312 eligible young patients with chondrosarcoma of bone. Table 1 summarized the detailed clinicopathological characteristics of all cases. The majority of patients were white (83.1%). The male to female ratio was 1.4–1.0.

**Table 1:** Baseline characteristics of 1312 young patients with chondrosarcoma of bone.

Variable	Value
Race	
White	1,090 (83.1%)
Black	126 (9.6%)
Others	96 (7.3%)
Gender	
Female	537 (40.9%)
Male	775 (59.1%)
Mean age (years)	28.5 ± 0.2
Year of diagnosis	
<2,000	471 (35.9%)
≥2,000	841 (64.1%)
Tumor site	
Appendicular	671 (51.1%)
Vertebral column	63 (4.8%)
Pelvic bones, sacrum, coccyx	230 (17.5%)
Rib, sternum and clavicle	163 (12.4%)
Others	185 (14.1%)
Tumor grade	
Low grade	937 (71.4%)
High grade	135 (10.3%)
Unknown	240 (18.3%)
Tumor subtype	
Chondrosarcoma-NOS	1,151 (87.7%)
Others	161 (12.3%)
Distant	
No	1,165 (88.8%)
Yes	75 (5.7%)
Unknown	72 (5.5%)
Tumor size (cm)	
<5	340 (25.9%)
5–10	344 (26.2%)
>10	175 (13.3%)
Unknown	453 (34.5%)
Surgery	
Yes	1,207 (92.0%)
No	105 (8.0%)
Radiotherapy	
Yes	155 (11.8%)
None/Unknown	1,157 (88.2%)
Chemotherapy	
Yes	137 (10.4%)
No/Unknown	1,175 (89.6%)
Marital status	
Married	533 (40.6%)
Others	721 (55.0%)
Unknown	58 (4.4%)
Dead	
Yes	267 (20.4%)
No	1,045 (79.6%)
5-year OS rate	88.50%
5-year CSS rate	89.10%

OS, overall survival; CSS, cancer-specific survival; NOS, means not otherwise specified.

The average age at diagnosis was 28.5 ± 0.2 years old (ranging from 1 to 40 years). Patients diagnosed before 2000 accounted for 35.9%, and patients diagnosed after 2000 accounted for 64.1%. 51.1% of cases were located in the extremity, 4.8% of cases in the vertebral column, 17.5% of cases in the pelvic bones, sacrum, coccyx, 12.4% of cases in rib, sternum and clavicle, and 14.1% of cases in other sites. More than two-thirds of patients (71.4%) were high grade. In addition, nearly 90% of cases of represented the Chondrosarcoma, NOS subtype. Only 75 patients (5.5%) presented with distant metastasis at diagnosis. Tumor size were divided into four groups: <5 cm (25.9%), 5–10 cm (26.2%), >10 cm (13.3%), and unknown (34.5%). The majority of the patients (92.0%) received surgery, only 11.8% of the patients received radiotherapy, and only 10.4% of patients received chemotherapy. Married patients accounted for 40.6%. At the time of data collection, 267 (20.4%) patients died. Kaplan–Meier survival results revealed that the 5-year OS and CSS rates of this cohort were 88.5% and 89.1%, respectively.

### Univariate cox regression analysis

Results of univariate analysis of OS and CSS were presented in Table 2. Univariate survival analysis revealed that risk factors including gender, year of diagnosis, tumor site, tumor grade, tumor subtype, distant metastasis, tumor size, surgery, radiotherapy, and chemotherapy had significant effects on both OS and CSS. As shown in univariate analysis, race and marital status had no effect on survival.

**Table 2:** Univariate Cox analysis of variables in young patients with chondrosarcoma of bone.

Variable	OS (*n* = 1312)		CSS (*n* = 1239)	
	HR (95% CI)	*p* value	HR (95% CI)	*p* value
Race				
White	1		1	
Black	1.257 (0.853–1.852)	0.247	1.375 (0.888–2.129)	0.153
Others	0.787 (0.459–1.351)	0.386	0.804 (0.436–1.481)	0.484
Gender				
Female	1		1	
Male	1.354 (1.051–1.745)	**0.019**	1.355 (1.007–1.824)	**0.045**
Year of diagnosis				
<2,000	1		1	
≥2,000	0.708 (0.539–0.929)	**0.013**	0.580 (0.431–0.781)	**<0.001**
Tumor site				
Appendicular	1		1	
Vertebral column	2.714 (1.729–4.259)	**<0.001**	3.456 (2.076–5.754)	**<0.001**
Pelvic bones, sacrum, coccyx	2.323 (1.732–3.116)	**<0.001**	3.102 (2.202–4.371)	**<0.001**
Rib, sternum and clavicle	1.039 (0.677–1.595)	0.86	1.156 (0.687–1.945)	0.585
Others	1.402 (0.952–2.063)	0.087	1.566 (0.995–2.465)	0.052
Tumor grade				
Low grade	1		1	
High grade	4.722 (3.477–6.413)	**<0.001**	5.952 (4.208–8.420)	**<0.001**
Tumor subtype				
Chondrosarcoma-NOS	1		1	
Others	1.799 (1.294–2.501)	**<0.001**	1.904 (1.329–2.727)	**<0.001**
Distant metastasis				
No	1		1	
Yes	6.949 (5.001–9.654)	**<0.001**	8.450 (5.954–11.991)	**<0.001**
Tumor size (cm)				
<5	1		1	
5–10	1.395 (0.869–2.239)	0.168	1.743 (0.997–3.047)	0.051
>10	5.038 (3.233–7.852)	**<0.001**	6.380 (3.766–10.807)	**<0.001**
Surgery				
No	1		1	
Yes	0.339 (0.243–0.473)	**<0.001**	0.283 (0.197–0.406)	**<0.001**
Radiotherapy				
None/Unknown	1		1	
Yes	2.101 (1.535–2.874)	**<0.001**	2.507 (1.791–3.511)	**<0.001**
Chemotherapy				
No/Unknown	1		1	
Yes	5.986 (4.618–7.760)	**<0.001**	9.103 (6.798–12.190)	**<0.001**
Marital status				
Married	1		1	
Others	1.198 (0.936–1.533)	0.151	1.243 (0.930–1.661)	0.141

OS, overall survival; CSS, cancer-specific survival, significant *p* value < 0.05. Unknown valuables were not shown. Values in bold mean they are statistically significant.

### Multivariate cox regression analysis

**Figure 2:**
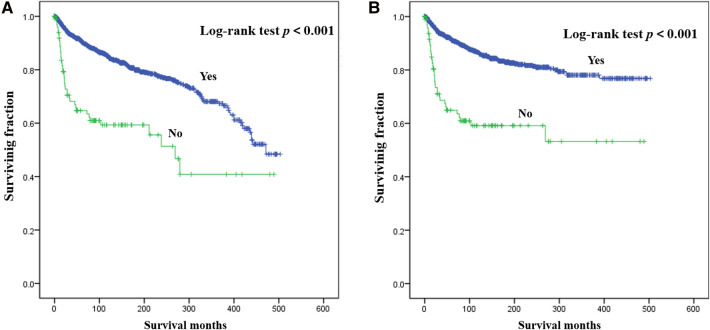
Kaplan-Meier method estimated OS (**A**) and CSS (**B**) in young patients with chondrosarcoma of bone stratified by surgery. OS, overall survival; CSS, cancer-specific survival.

**Figure 3:**
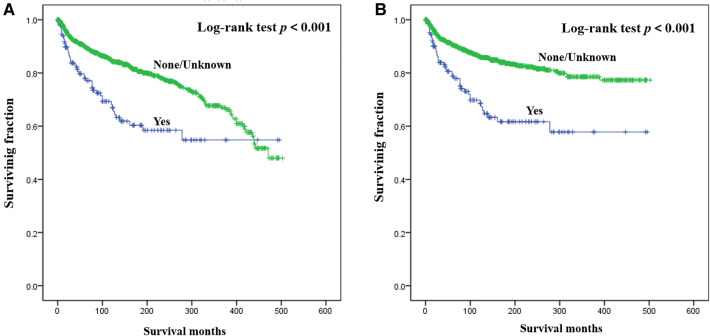
Kaplan-Meier method estimated OS (**A**) and CSS (**B**) in young patients with chondrosarcoma of bone stratified by radiotherapy. OS, overall survival; CSS, cancer-specific survival.

**Figure 4:**
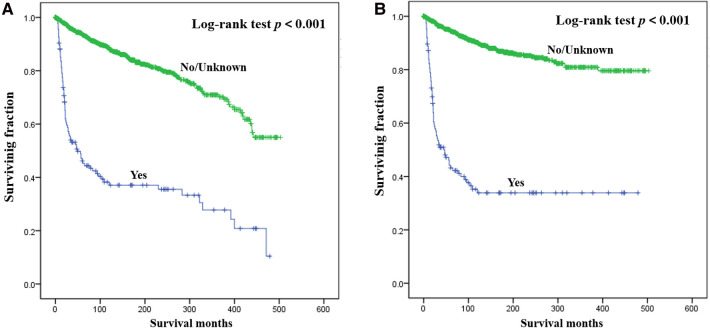
Kaplan-Meier method estimated OS (**A**) and CSS (**B**) in young patients with chondrosarcoma of bone stratified by chemotherapy. OS, overall survival; CSS, cancer-specific survival.

Results of multivariate survival analysis of these patients were shown in Table 3. Multivariate Cox analysis showed gender, year of diagnosis, tumor site, tumor grade, distant metastasis, tumor size, surgery, and chemotherapy were independent indicators of poor prognosis. Tumor subtype was identified as an independent predictor of CSS (*p* < 0.05), but not an independent risk factor of CSS (*p* > 0.05). Radiotherapy was no longer statistically significant in multivariate analysis. We also generated Kaplan-Meier curves to intuitively show the survival difference stratified by different treatments. As shown in Figure 2, patients who underwent surgery had significantly improved OS and CSS. However, the prognosis of patients who received radiotherapy (Figure 3) or chemotherapy (Figure 4) was significantly reduced.

**Table 3:** Multivariate Cox analysis of variables in young patients with chondrosarcoma of bone.

Variable	OS (*n* = 1312)		CSS (*n* = 1239)	
	HR (95% CI)	*p* value	HR (95% CI)	*p* value
Gender				
Female	1		1	
Male	1.368 (1.049–1.784)	**0.021**	1.441 (1.050–1.978)	**0.024**
Year of diagnosis				
<2,000	1		1	
≥2,000	0.708 (0.522–0.961)	**0.027**	0.600 (0.427–0.842)	**0.003**
Tumor site				
Appendicular	1		1	
Vertebral column	2.072 (1.271–3.378)	**0.003**	2.431 (1.385–4.266)	**0.002**
Pelvic bones, sacrum, coccyx	1.909 (1.399–2.605)	**<0.001**	2.467 (1.716–3.546)	**<0.001**
Rib, sternum and clavicle	1.248 (0.803–1.941)	0.325	1.293 (0.754–2.216)	0.35
Others	1.305 (0.849–2.006)	0.225	1.299 (0.790–2.136)	0.303
Tumor grade				
Low grade	1		1	
High grade	2.858 (1.937–4.217)	**<0.001**	3.187 (2.035–4.989)	**<0.001**
Tumor subtype				
Chondrosarcoma-NOS	1		1	
Others	0.719 (0.486–1.062)	0.097	0.568 (0.367–0.880)	**0.011**
Distant metastasis				
No	1		1	
Yes	3.468 (2.352–5.115)	**<0.001**	4.209 (2.733–6.483)	**<0.001**
Tumor size (cm)				
<5	1		1	
5–10	1.029 (0.634–1.668)	0.909	1.170 (0.660–2.072)	0.591
>10	3.114 (1.924–5.042)	**<0.001**	3.319 (1.866–5.902)	**<0.001**
Surgery				
No	1		1	
Yes	0.502 (0.343–0.736)	**<0.001**	0.572 (0.369–0.885)	**0.012**
Radiotherapy				
None/Unknown	1		1	
Yes	1.123 (0.775–1.628)	0.539	1.143 (0.766–1.704)	0.514
Chemotherapy				
No/Unknown	1		1	
Yes	2.620 (1.850–3.711)	**<0.001**	3.539 (2.359–5.309)	**<0.001**

OS, overall survival; CSS, cancer-specific survival, significant *p* value <0.05. Unknown valuables were not shown. Values in bold mean they are statistically significant.

## Discussion

Young patients with chondrosarcoma are a rare cancer population. Previous studies seldom documented their clinicopathological characteristics and risk factors of survival. Given that age is an important risk factor for the prognosis of chondrosarcoma, outcome analysis in different age subgroups should be made whenever possible. To the best of our knowledge, this is the first presentation of young patients (age less than 40 years old) with chondrosarcoma of bone. In the current study, young patients with chondrosarcoma of bone experienced prolonged survival, with the 5-year OS and CSS rates 88.5% and 89.1%, which were better than that of all chondrosarcomas (10, 11). Elderly patients may have a higher probability of pulmonary metastasis (12). However, some studies have reported poorer (13) or similar prognosis (14, 15) in young patients with chondrosarcoma than in older patients. Moreover, nine independent predictors of prognosis were identified, including gender, year of diagnosis, tumor site, tumor grade, tumor subtype, distant metastasis, tumor size, surgery, and chemotherapy, which is very helpful for decision-making.

In the present study, we found that male patients had worse OS and CSS than female patients, which was consistent with that of other researches (9, 12). Patients diagnosed after 2000 were significantly correlated with increased OS and CSS, suggesting advances in treatment and diagnosis of chondrosarcoma. We demonstrated that tumor size, tumor grade, and metastatic status were independent prognostic factors for survival, which was confirmed by many other researches (9, 16). With regard to tumor site, chondrosarcomas in spine or pelvis were correlated with poorer survival. Some studies reported that marital status was as an independent risk factor for survival of chondrosarcoma (17, 18). However, our univariate analysis showed that marital status did not impact patients' survival. Further research is needed to confirm this finding.

Few treatment options exist for chondrosarcoma. Typically, chondrosarcomas are insensitive to radiotherapy and chemotherapy (19). Some studies showed that radiotherapy could decrease chondrosarcoma recurrence (20), whilst other studies indicated it could benefit the survival of chondrosarcoma (17). Armin Arshi et al. (21) found that radiotherapy prolonged survival in patients with metastatic disease and worsened survival in patients with local disease. Kun-Chi Hua et al. (4) reported that radiotherapy or chemotherapy had no effect on the prognosis of all chondrosarcoma. Our study also found that radiotherapy was not an independent survival predictor. It is worth noting that radiation-induced chondrosarcomas were reported (22, 23). Interestingly, young patients with chondrosarcoma of bone who received chemotherapy experienced worse OS and CSS than those no/unknown chemotherapy. Conventional chondrosarcoma does not respond to chemotherapy (24). Additionally, chemotherapy was not associated with improved survival (16, 25). Only surgery can provide survival benefits for young patients with chondrosarcoma of bone, which was in line with other studies on chondrosarcoma (16, 26, 27). Zhan Wang et al. (28) reported that surgical treatment of primary tumors could increase the survival of metastatic chondrosarcoma. Our study may provide an update on current and future treatment options for young chondrosarcomas.

This study has some shortcomings. This study has retrospective nature, which might generate potential selection bias. Second, some risk factors were not available in the SEER database, such as detailed treatment methods and recurrence information during the follow-up. Additionally, the SEER database does not provide a clear Yes/No option for chemotherapy and radiotherapy. Nevertheless, the SEER database makes it possible to study rare cancer populations, such as the young chondrosarcoma population in this study.

## Conclusion

Young patients with chondrosarcoma of bone presented a relatively prolonged survival, with the 5-year OS and CSS rates 88.5% and 89.1%. This study identified nine meaningful prognostic factors in this special entity, including gender, year of diagnosis, tumor site, tumor grade, tumor subtype, distant metastasis, tumor size, surgery, and chemotherapy. Based on our findings, we recommend surgical treatment for young patients with chondrosarcoma of bone and do not recommend chemotherapy for these patients. More importantly, the role of chemotherapy and radiotherapy among these patients should be clarified in the future.

## Data Availability

The raw data supporting the conclusions of this article will be made available by the authors, without undue reservation.
